# Modeling of the HIV epidemic and continuum of care in French Guiana

**DOI:** 10.1371/journal.pone.0197990

**Published:** 2018-05-24

**Authors:** Mathieu Nacher, Leila Adriouch, Florence Huber, Vincent Vantilcke, Félix Djossou, Narcisse Elenga, Antoine Adenis, Pierre Couppié

**Affiliations:** 1 Centre d’Investigation Clinique Antilles Guyane, INSERM 1424, Centre Hospitalier de Cayenne, Cayenne, French Guiana; 2 COREVIH Guyane (Coordination de la lutte contre le VIH), Centre Hospitalier de Cayenne, Cayenne, French Guiana; 3 Service de Médecine, Centre Hospitalier de l’Ouest Guyanais, Saint Laurent du Maroni, French Guiana; 4 Department of Infectious and Tropical Diseases, Centre Hospitalier de Cayenne, Cayenne, French Guiana; 5 Department of Pediatrics, Centre Hospitalier de Cayenne, Cayenne, French Guiana; 6 Department of Dermatology, Centre Hospitalier de Cayenne, Cayenne, French Guiana; The Ohio State University, UNITED STATES

## Abstract

**Background:**

In order to compute the continuum of care for French Guiana, it is necessary to estimate the total number of persons living with HIV. The main objective was to determine how many persons were infected with HIV and how many were unaware of it.

**Methods:**

We used 2 different models to calculate the total number of persons infected with HIV: Spectrum’s AIM module using CSAVR to compute incidence from case registration and vital statistics; and the ECDC model from the French Guiana HIV cohort data.

**Result:**

The present results show that both models led to similar results regarding the incident number of cases (i.e. for 2016 174 versus 161) and the total HIV population (in 2016 3206 versus 3539) respectively. The ECDC modeling tool showed that the proportion of undiagnosed HIV infections declined from 50% in 1990 to 15% in 2015. This amounted to a stable or slightly increasing total number of undiagnosed patients of 520.

**Conclusions:**

The estimations of the total HIV population by both models show that the HIV population is still growing. The incidence rate declined in 2000 and the decline of the number of newly acquired HIV infections, after a decline after 2003 is offset by population growth. The proportion of undiagnosed infections has declined to 15% but the number of undiagnosed infections remains stable. The HIV cascade shows that despite good results for treatment in care, reaching the 90*90*90 UNAIDS target may be difficult because a significant proportion of patients are lost to follow-up.

## Introduction

The 2020 objective for UNAIDS is the 90*90*90 cascade of care with 90% of the HIV population aware of their diagnosis, 90% of these being on antiretrovirals, and 90% of those on antiretrovirals having virological suppression[1]. Overall, in this framework, 73% of all HIV patients should thus be virologically suppressed. To obtain this cascade of care it is necessary to estimate the total number of patients living with HIV. This requires modeling based on surveillance data. French Guiana is the French territory most affected by the HIV epidemic. The epidemic mostly affects heterosexual migrants and is driven by multiple sexual partnerships sex work and crack use[2]. The health system is that of France with free access to care and treatment, and residence papers for foreigners from less developed countries. With new antiretrovirals and the universal treatment strategy, mortality and AIDS have greatly declined, and retention in care has increased[3]. However, there has never been any formal evaluation of the total number of persons living with HIV (persons aware and unaware of their HIV diagnosis). The objective of the present study was to estimate this number, and HIV incidence using 2 different modeling tools: UNAIDS's SPECTRUM (AIM/CSAVR)[4] and ECDC's HIV modeling tool[5].

## Methods

### Case surveillance and vital registration data

Crude HIV surveillance data for France and French Guiana is freely available on the web site of the French National Public Health Agency (Santé Publique France)[6,7]. This data has been collected since 2003 but is aggregated until 2007 when it is available on a yearly basis. Briefly laboratories finding a positive HIV test must fill a mandatory reporting form (or recently an online declaration) where the patient identity is anonymized with specific software from Santé Publique France, and sent to the prescriber of the HIV test to fill demographical, epidemiological and clinical aspects, and then sent to regional health agencies which then transmit the data to the national level where duplicates are removed and national statistics are produced[8,9].

### HIV programme data

Data on HIV patients in French Guiana has been available since 1989. It is independent from the above case surveillance and vital registration data. Clinical, biological and epidemiological data was enteredin the DMI2 government programme until 2008, and in eNADIS/DATAIDS since then by specific trained research technicians as described elsewhere.[10,11]

This allowed us to obtain the number and proportion of patients lost to follow up (not seen in more than 12 months), the proportion of patients on ARV in 2016, and the virological suppression rate in those on ARV. These figures were applied to the HIV total population estimates (diagnosed and undiagnosed) to obtain the overall proportion of patients that were virologically suppressed in 2016.

### First model

Spectrum version 5 (v5,51 beta 23) was used using the AIM module (AIDS Impact Model) [12]and the CSAVER (Case Surveillance and Vital Registration) incidence fitting tool with a start in 1970 and projections until 2021. Historical HIV programme data was entered in the file with treatment eligibility criteria for adults and children for different periods;data on the proportion of pregnant women with access to prevention of mother to child transmission of HIV; number of males and females receiving ARV, the median CD4 countat antiretroviral initiation, the proportion of treated patients that were virologically suppressed; and the proportion of lost to follow up patients each year. For 2016, the patients lost to follow up were defined as not seen for over a year (last seen before 2015). These data were obtained from the eNADIS and DMI2 computerized medical records that are part of the French Hospital Database for HIV.

The annual rate of progression to the next CD4 category, HIV mortality with and without HAART were selected from the options in spectrum based on Latin America and the Caribbean. The default ratio of fertility of infected versus uninfected women was chosen. The Epidemic was modeled as a concentrated epidemic.

Incidence was fitted using the yearly HIV mandatory reporting surveillance data from Santé Publique France (formerly the Institut National de Veille Sanitaire (INVS)), which is available online between 2007 and 2015[6]. HIV/AIDS deaths from 2001 and 2014 were obtained from the CEPIDC-INSERM, which collects and studies all death certificates in France and its overseas territories[7]. A custom ratio for male to female incidence was set to reflect the sex ratio in our cohort, missing values were linearly interpolated from available data.

CSAVER (Case Surveillance and Vital Registration) was used to fit incidence to the data using maximum likelyhood estimates. The final model had an alpha (rate of increase at the beginning of the trend) of 0.65633, a beta (rate of convergence to the asymptote) of 0.07089, t0 (the time of the inflexion point) at 1997.73744, a (peak incidence value) 0.1577 and b (asymptote) at 0.04802.

### Second model

ECDC HIV modeling tool v1.2.2 was also used to model the HIV epidemic using different data sources. Briefly, this multistate back-calculation model estimates HIV incidence, time between infection and diagnosis, and the undiagnosed population by CD4 count strata, using surveillance data on new HIV and AIDS diagnoses[13]. Data from the HIV cohort (French Hospital Database on HIV) between 1989 and 2016 was used. The annual number of new HIV diagnoses, the annual number of AIDS cases and the annual number of new HIV cases with a concurrent AIDS diagnosis were used as separate CSV files to be uploaded in the HIV modeling tool. The time intervals used were 1980–1984, 1984–1996, 1996–2004, 2004–2008, and 2008–2016 starting from the new baseline at the end of each interval.

#### Ethical and regulatory aspects

Patients included in the FHDH gave written informed consent for the use of their data. Their identity is encrypted before sending the data to the Institut National de la Recherche Médicale (INSERM) which centralizes data from Regional Coordination for the fight against HIV (COREVIH) throughout France. This cohort was approved by the Commission Nationale Informatique et Libertés (CNIL) since Nov 27th 1991 and has led to numerous international scientific publications.

## Results

**Fig 1:**
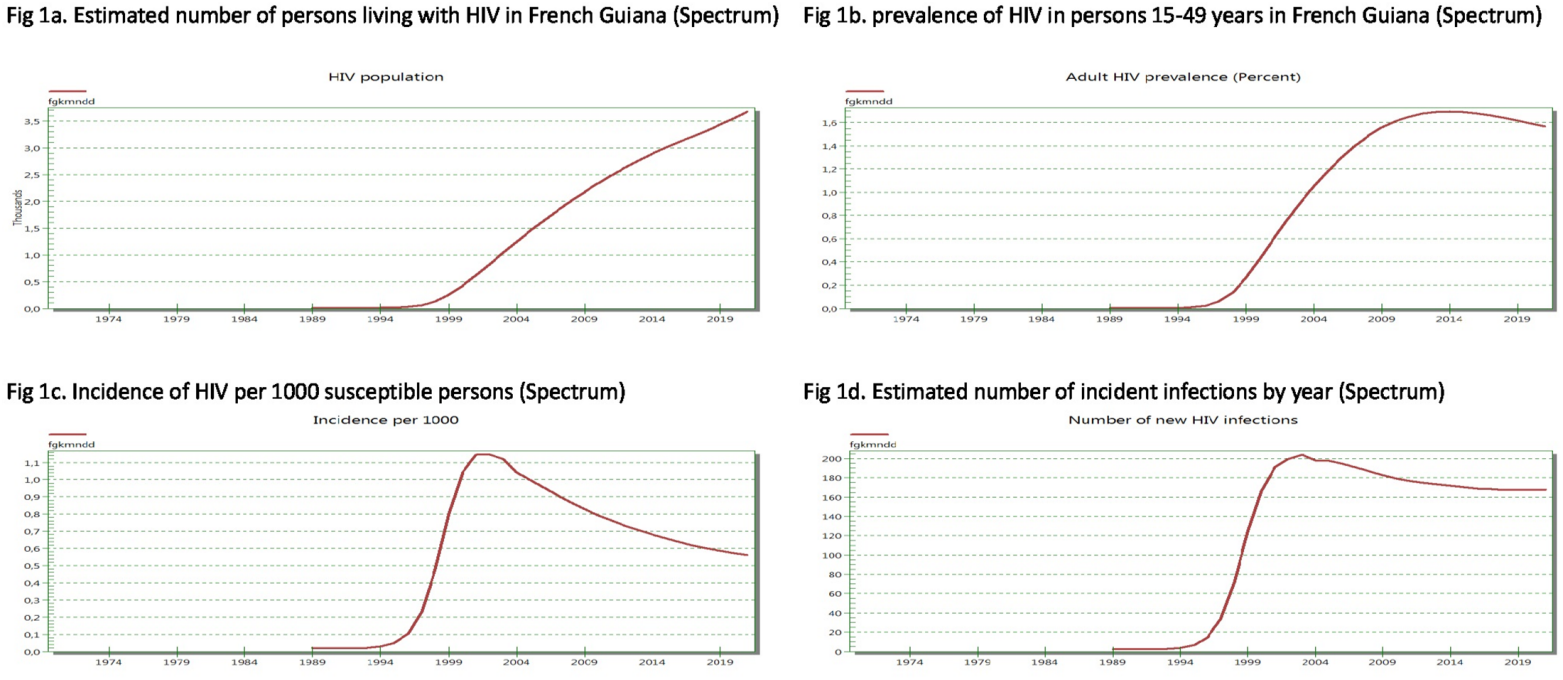
Spectrum modeling of total numbers of HIV-infected persons, prevalence, incidence rate, and number of incident infections in French Guiana. (a) Estimated number of persons living with HIV in French Guiana (Spectrum); (b) prevalence of HIV in persons 15–49 years in French Guiana (Spectrum); (c) Incidence of HIV per 1000 susceptible persons (Spectrum); and (d) Estimated number of incident infections by year in French Guiana (Spectrum).

**Fig 2:**
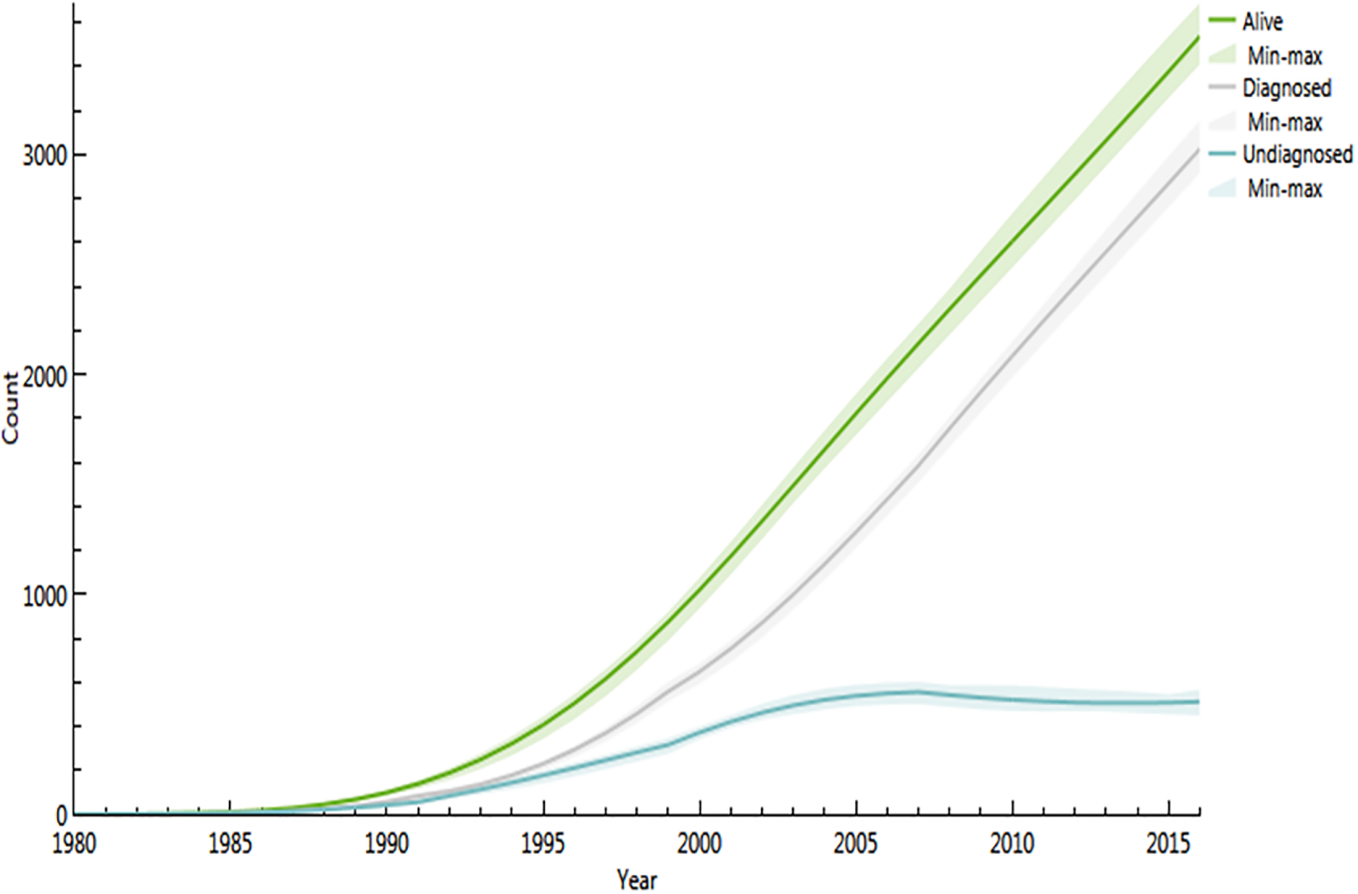
Total estimated number of persons living with HIV in French Guiana by year with confidence intervals using the ECDC HIV modeling tool.

**Fig 3:**
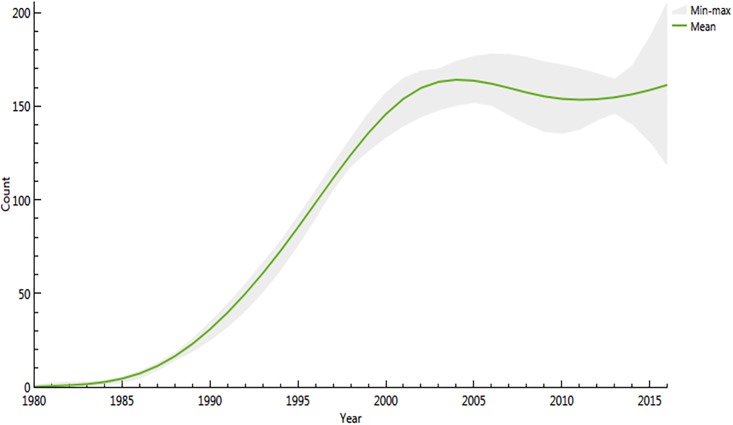
Estimated number of incident infections by year in French Guiana with confidence intervals using the ECDC HIV modeling tool.

The Spectrum model is shown in Fig 1a, 1b, 1c and 1d and the ECDC model is shown in Figs 2 and 3. The present results show that both models led to similar results regarding the total HIV population (in 2016 3206 versus 3539) and the incident number of cases (i.e. for 2016 174 versus 161) respectively Figs 1a, 2, 1d and 3.

Among these, the ECDC modeling tool showed that the proportion of undiagnosed HIV infections declined from 50% in 1990 to 15% in 2016. This amounted to a stable or slightly increasing total number of undiagnosed patients of 520 versus 2861 diagnosed HIV infections. The estimated delay between infection and diagnosis in 2016 was 3.2 years. Using Spectrum/CSAVR, the incidence rate estimations declined from a maximum rate of nearly 1.15 per 1000 persons at risk in 2001 to 0.65 per 1000 persons at risk in 2016 and a prediction of 0.55 in 2020 (Fig 1c). The estimated adult prevalence in adults 15 to 49 was slightly declining at 1.7% in 2015 and prevalence in pregnant women was estimated at 1.3%, very close to what is actually measured in the main 3 maternity wards in French Guiana. The estimated HIV population aged 1–14 was estimated to be at 42, which is very similar to the actual number of children followed in French Guiana (21 in Cayenne, 20 in Saint Laurent du Maroni and 5 in Kourou, (Source Dr Elenga, Head, Department of Pediatrics)).

The ECDC modeling tool estimated the median delay between infection and diagnosis to be 3.2 years (95% CI 3.03–3.4).

### Estimated impact

The estimated impact of antiretroviral therapy in French Guiana (comparing with a scenario where no one would receive antiretrovirals) suggested that in 2015 alone 95 lives were saved by ARVs, in children (0–4 years) 7 deaths were avoided by the prevention of mother to child transmission and 4 by ARVs. Finally, over 20 transmissions from mother to child are prevented each year, 23 in 2016. Overall, it was estimated using AIM/SPECTRUM that, in 2016 alone, ARVs and PMTCT saved over 1200 life years.

## Continuum of care

Table 1 shows the continuum of care in 2016. Overall, when taking into account the undiagnosed and the lost to follow up, 57.8% of all HIV patients at the time were virologically suppressed (viral load <20 copies per ml). It is noteworthy that overall 17.8% of patients were lost to follow up with a marked reduction (from a median number of 56 per year (IQR = 49–72) to 24) of the number lost to follow up by year in 2016 (last seen for more than 12 months)the year when universal treatment started to be systematically implemented.

**Table 1:** Continuum of care of HIV infection in French Guiana, 2016.

Estimated number of PLWHIV*	HIV prevalence**	Proportion undiagnosed	Proportion lost to follow-up (>12 months)	Proportion in care on on ART	Proportion on ART &virologically suppressed	Proportion of all PWLHIV (diagnosed and undiagnosed)virologicallysuppressed***
3206–3539	1.18%-1.35%	15%	17.8%	91%	91%	57.8%

*SPECTRUM-ECDC models;

**total population 262 527;

*** Obtained by multiplying proportion diagnosed X proportion on ARV X proportion virologically suppressed.

## Discussion

Globally estimates from the 2 models based on different data sources were fairly consistent in terms of total HIV population and number of new HIV infections per year. In 2016, according to the UNAIDS objectives, French Guiana was thus at 85% x 74.8% x 91%. Given the proportion of patients lost to follow up, the proportion of all HIV-infected patients (both diagnosed and undiagnosed) that were virologically suppressed was 57.8% which is still far from the 73% target. A recent study showed that in mainland France the overall proportion of virologically suppressed patients was 72% but the calculations did not account for those lost to follow-up[14]. Using the same calculation methods for French Guiana would lead to a 70% of all HIV patients virologically suppressed, very close to the target but omitting a major challenge of HIV care: retention in care. Since the systematic implementation of universal antiretroviral treatment, there has been a drop in the number of follow-up interruptions suggesting that ARV treatment is an incentive to come to the HIV clinic, as observed in a previous study in French Guiana[15]. It is however too early to determine if this trend will persist in future years.

The incidence of HIV peaked in the early 2000s and has gradually declined since then. This presumably reflects the benefits of virological suppression on HIV-transmission, and possibly a saturation of the reservoirs of the most at risk populations. The estimated number of new infections peaked in 2003 and may have remained stable or slightly declined since then despite the continuous increase of the number of persons living with HIV. This relative disconnection between trends forincidence rate and infection numbers is linked to the very high population growth rate in French Guiana, where incidence rate decline may be offset by the demographic growth of the population[16,17]. Although the numbers for a given year were close (15 person difference), the ECDC model estimates showed a slight increase with large confidence intervals in the 3 last years, whereas the CSAVR model showed a slow regular decrease in new infection numbers. The estimated number of new infections should not be taken literally given the apparent differences of the 2 models, appending additional data in the future may resolve this discrepancy.

Despite the significant decline of the proportion of patients unaware of their diagnosis, the total number seemed stable which is consistent with what we have observed in French Guiana with 30% of new diagnoses concerning patients with advanced HIV disease despite great efforts to reduce this proportion[18,19] and delayed access to specialized care[20]. Others have also shown that initial progress in reducing the proportion of patients with advanced diseasewas followed by a plateau of advanced diagnoses with no further improvement[21]. This group should thus be a core priority if we wish to make further progress on transmission and on morbidity and mortality.

For the Spectrum model, we used CSAVR to fit incidence because although there is cohort data on HIV patients, although epidemiologic surveillance and vital registration statistics are available, there is a striking lack of seroprevalence surveys in most at risk populations which precluded us from modeling the epidemic using specific populations driving the epidemic. Perhaps the fact that the epidemic is often conceptualized as generalized in French Guiana has not helped looking more precisely at risk group serological surveillance.[2] The estimates are obtained by models which may have some imprecision and which depend on the quality of the data inputs. French Guiana having 3 main centers of HIV care, and a limited number of laboratories it is arguable that the task of compiling data may be easier than in large cities with numerous laboratories and HIV clinics. In French Guiana, the epidemic is very particular because over 75% of patients are migrants, a situation resulting from the High GDP per capita of French Guiana relative to the rest of Latin America. This “open system” may thus introduce inaccuracies in estimations based on model assumptions based on closed epidemic dynamics. Although, over half of foreign patients are estimated to acquire HIV in French Guiana,[22] in 2015–2016 massive population fluxes may lead the model estimates to underestimate the actual number of persons with HIV.

In conclusion, the estimations of the total HIV population by both models were fairly in agreement, incidence has started to decline in 2000 and the number of new HIV infections in 2003 but this may have been offset by population growth. The proportion of undiagnosed infections has declined to 15% but the number of undiagnosed infections remains stable. Overall, when looking at the proportion of diagnosed patients, the proportion on treatment and the proportion that were virologically suppressed, the continuum of care in French Guiana was similar to what was reported in France. However, including the interruption of care markedly impacted the overall proportion of virologically suppressed patients in French Guiana. Overall, this gives a picture of the epidemic in French Guiana and reemphasizes the need and the challenge of testing patients early and retaining them in care, which is plausibly more challenging than in mainland France given the large proportion of socially precarious patients.
